# Effect of Chromosome Tethering on Nuclear Organization in Yeast

**DOI:** 10.1371/journal.pone.0102474

**Published:** 2014-07-14

**Authors:** Barış Avşaroğlu, Gabriel Bronk, Susannah Gordon-Messer, Jungoh Ham, Debra A. Bressan, James E. Haber, Jane Kondev

**Affiliations:** 1 Department of Physics, Brandeis University, Waltham, Massachusetts, United States of America; 2 Department of Biology, Brandeis University, Waltham, Massachusetts, United States of America; 3 Rosenstiel Basic Medical Sciences Research Center, Brandeis University, Waltham, Massachusetts, United States of America; 4 Department of Biochemistry, Brandeis University, Waltham, Massachusetts, United States of America; Cancer Research UK London Research Institute, United Kingdom

## Abstract

Interphase chromosomes in *Saccharomyces cerevisiae* are tethered to the nuclear envelope at their telomeres and to the spindle pole body (SPB) at their centromeres. Using a polymer model of yeast chromosomes that includes these interactions, we show theoretically that telomere attachment to the nuclear envelope is a major determinant of gene positioning within the nucleus only for genes within 10 kb of the telomeres. We test this prediction by measuring the distance between the SPB and the silent mating locus (*HML*) on chromosome III in wild–type and mutant yeast strains that contain altered chromosome-tethering interactions. In wild-type yeast cells we find that disruption of the telomere tether does not dramatically change the position of *HML* with respect to the SPB, in agreement with theoretical predictions. Alternatively, using a mutant strain with a synthetic tether that localizes an *HML*-proximal site to the nuclear envelope, we find a significant change in the SPB-*HML* distance, again as predicted by theory. Our study quantifies the importance of tethering at telomeres on the organization of interphase chromosomes in yeast, which has been shown to play a significant role in determining chromosome function such as gene expression and recombination.

## Introduction

### Chromosome organization during interphase

Many different lines of experimental evidence have revealed that chromosomes in cells are organized in space and in time [1]–[4], and that this organization has a strong influence on chromosome functions such as gene expression, DNA-damage repair, recombination, and replication [4]–[9]. Genome-wide studies that have addressed long-range chromatin interactions over the past decades suggest a non-random organization of eukaryotic chromosomes during interphase [10]–[15]. The idea of chromosome territories has emerged whereby chromosomes are segregated and occupy specific non-overlapping sub-regions of the nucleus [16].

While distinct chromosome territories exist in the nucleus of higher eukaryotes [11], [14], [17], [18], a highly intermingled yet polarized arrangement of chromosomes is prominent in the interphase nucleus of budding yeast, *Saccharomyces cerevisiae* [12], [15], [19]. Rabl was the first to describe this arrangement of chromosomes in salamander larvae cells in 1885 [20]. Its most prominent feature is the attachment of chromosomes at the nuclear envelope in a polarized fashion [21]. Specifically, in budding yeast centromeres of all the chromosomes are attached via microtubules to the spindle pole body (SPB), which is a large protein complex in the nuclear envelope [22]–[24]. Chromosomes during interphase are also tethered to the nuclear periphery at their telomeres through protein pathways that involve Yku70, Yku80, Sir4, Esc1, Mps3, and Siz2 [25]–[29].

Another major feature of non-random chromosome organization in yeast is the clustering of ribosomal DNA at the pole of the nucleus opposite the SPB, resulting in the nucleolus [12], [30]–[32]. The nucleolus seems to exclude other genetic loci from the region of the nucleus that it occupies. The modern version of the Rabl model of nuclear organization takes into account the effects of chromosome tethering and volume exclusion by the nucleolus, and it provides a basis for studying long-range DNA interactions in the yeast nucleus [15], [19], [33]–[36].

Tethering of genes to the nuclear periphery can affect their function. Namely, genes that are localized to the nuclear periphery can be repressed [37], [38] or in some instances activated [8], [39], [40], while in the context of DNA damage repair, disruption of tethering interactions can affect repair machinery [38], [41]. Even though multiple studies have underscored the functional importance of tethering interactions, we are still lacking a quantitative understanding of the interplay between chromosome tethering and the spatial positioning of genes within the nucleus. This study seeks to remedy that situation.

### Polymer model of yeast chromosome organization

At length scales of tens of nanometers DNA in the nucleus is wrapped around histones to form nucleosomes [42] which can be packed into the chromatin fiber in a number of different arrangements [10], [43], [44]. Despite this structural complexity at small scales, on larger length scales corresponding to hundreds of nanometers, a number of experimental studies of chromosome organization in different types of cells have suggested that chromosomes can be modeled as polymers characterized by two material parameters: the persistence length and the DNA packing density [45]–[47]. For budding yeast the emerging consensus is that the large scale mechanical properties of chromosomes are well described by a polymer model with a persistence length of approximately 100nm and a packing density of 25 bp per nanometer of chromatin fiber [13], [48] (for comparison, the persistence length of naked DNA is 50 nm [49], [50] and it has a packing density is 3 bp/nm [51]). An implicit assumption being made here is that equilibrium polymer models can be used to describe interphase chromosomes in yeast. Indeed, measurements of chromosome dynamics [2], [52] and simple theoretical estimates [53]–[60] are both consistent with the idea that interphase chromosomes in yeast can be regarded as being in equilibrium on the time scales set by the cell cycle (approximately 2 hrs).

The usefulness of a polymer model lies in its ability to predict the probability distribution of distances between any two genes on the yeast genome. When the genes are on the same chromosome and separated by more than a few persistence lengths, this probability is well approximated by a random-walk polymer model in which polymer configurations correspond to paths traced out by a random walker who makes steps equal to twice the persistence length (also known as the Kuhn length, 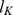) [61]. Given the estimates for the persistence length (100 nm) and the packing density (25 bp/nm), one Kuhn segment contains approximately 5 kb of DNA [62]. It is important to note that in the random-walk polymer model the probability distribution of physical distances between genes (in micrometers) as a function of their separation along the chromosome (in base-pairs) only depends on the ratio of the Kuhn length and the packing density, which we refer to as the extension parameter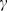 (in units of micrometers squared per mega base pairs). Therefore experiments that measure the distance distribution between genes that are separated by more than a few persistence lengths can be used to extract the extension parameter, but not the Kuhn length and the packing density separately.

Yeast chromosomes are confined to the nucleus, which is roughly spherical with a radius of about one micrometer. The haploid yeast genome is 2400 Kuhn segments long, which follows from the fact that the genome consists of 12 Mb of DNA, which are distributed over 16 chromosomes of varying length. Therefore the density of chromatin in the yeast nucleus is 600 Kuhn segments per cubic micron. This should be compared to the overlap concentration 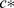 [61] which is the concentration that a typical yeast chromosome would have if it were released from the confining influence of the nucleus, 
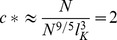
 Kuhn segments per cubic micron. For this estimate we used 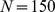 Kuhn segments (2400/16), which assumes a typical yeast chromosome length of 750 kb, and the formula for the volume occupied by a random-walk polymer of 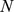 Kuhn segments, which is approximately 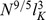 [61].

Given that the chromatin density in the yeast nucleus is more than two orders of magnitude greater than the overlap concentration (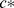) the Flory theorem should hold [53], [63]. Namely, this dense polymer system has the property that the self-avoiding interactions between Kuhn segments of the same polymer chain are screened by the presence of other chains that interpenetrate it. In this situation the statistics of individual chains are the same as that of an ideal random-walk polymer, which ignores self-avoidance of the Kuhn segments. We therefore model individual yeast chromosomes as ideal random-walk polymers.

In addition to this theoretical argument, results of recent chromosome conformation capture experiments on yeast chromosomes can also be used to justify the model of yeast chromosomes as ideal random-walk polymers (from now on referred to simply as “random-walk polymers”). Namely, a random walk of 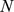
steps extends over a volume that grows as 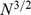 (as opposed to the 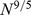 scaling that holds for self-avoiding random-walks). This implies a contact frequency between genes that scales as their separation along the chromosome to the power -3/2. Measurements by chromosome conformation capture of the contact frequency for pairs of genes on the same chromosome that are separated by distances between 30 and 500 kb (6 and 100 Kuhn segments) confirm the predicted power of -3/2 [15], [55].

Here we present theoretical calculations and quantitative experiments that address the role of telomere tethering on chromosome organization in the interphase nucleus of yeast cells. We use a random-walk polymer model of yeast chromosomes that incorporates volume exclusion by the nucleolus and tethering constraints consistent with Rabl organization. We extract the parameters that define our polymer model from three-dimensional distance measurements between a fluorescently tagged genetic locus proximal to *HML* and the fluorescently labeled SPB in wild type and mutant yeast cells, and find them to be in good agreement with previously reported values. Then, using the random-walk polymer model of chromosomes, we compute the effect of telomere tethering on the spatial locations of genes in the yeast nucleus. We find that only genes that are very close, within approximately 10 kb of the telomere have their positioning significantly affected by tethering. The effect of the tether decays with distance from the telomere exponentially with a characteristic length of 20 kb. We test our theoretical predictions against data from experiments on mutant cells that have either disrupted telomere tethering, or an additional tether at an *HML* proximal site, and find good agreement between theory and experiments.

## Results

### Tethering of yeast chromosomes at telomeres only affects the positioning of genetic loci close to the telomere

Clustering of centromeres around the SPB via microtubule attachments and tethering of telomeres to the nuclear periphery are the two major determinants of the Rabl-like organization of interphase chromosomes in the yeast nucleus. Here we investigate theoretically the extent to which tethering of chromosomes at the telomeres influences gene positioning within the interphase nucleus.

**Figure 1:**
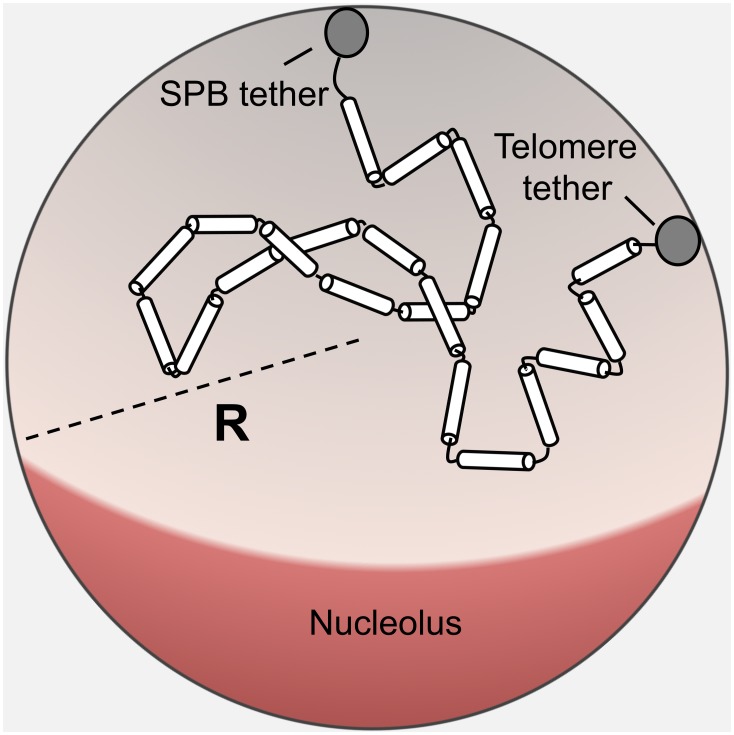
Random walk model of yeast chromosomes. A single arm of the yeast interphase chromosome is modeled as a random-walk polymer confined to a sphere of radius *R* and tethered at its ends to the surface of the sphere. The spindle pole body (SPB) tether (gray circle) is positioned at the north pole while the telomere tether (gray circle) is allowed to take any position on the surface of the sphere. The random walk polymer is made up of rigid segments of equal length (Kuhn length) connected by flexible links. In addition to spherical confinement, an impenetrable sub volume (red spherical cap) representing the nucleolar region limits the space available for the chromosome.

We model interphase chromosomes in the yeast nucleus as confined and tethered random walk polymers (Figure 1). A sphere of radius 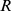 represents the nucleus, and the nucleolus is modeled by an impenetrable spherical-cap that occupies a fraction 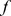
of the nuclear volume. The chromosome is made up of Kuhn segments that each consists of 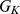 base pairs of DNA and each Kuhn segment is 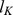 microns in length. A valid chromosome configuration is any path of a random walker that begins 50 nm away from the north pole (accounting for the microtubule that connects the centromere to the SPB [64], [65]) and ends at the surface of the sphere (this constraint accounts for telomere tethering) while remaining within the confines of the nucleus. The parameters of the polymer model (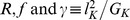; in Table 1) were extracted from our experiments that measure the position of a fluorescently labeled gene with respect to the SPB in the interphase yeast nucleus, using maximum likelihood estimation (MLE) (see Text S1).

**Table 1:** Model Parameters.

Parameter Name	Previously reported experimental values	Value used in the model (range tested in MLE)
Mean nuclear radius ( 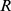 )	0.9–1.05 µm [12], [36], [74], [83]	0.95 µm (0.8–1.15 µm)
Standard deviation of nuclear radius	0.07–0.15 µm [12], [36]	0.09 µm (0.04–0.14 µm)
Nucleolar volume fraction ( 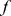 )	15–30% of the nuclear volume [12], [36], [84]	20% of the sphere volume of radius 0.95 µm (0–45%)
Chromosome extension parameter ( 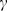 )	7–13 µm^2^/Mbp [13]	13 µm^2^/Mbp (7–13 µm^2^/Mbp)
SPB to centromere distance	50–300 nm [64], [65], [85]	50 nm (0–200 nm)
Telomere to nuclear envelope distance	Not measured	50 nm (0–50 nm)

**Figure 2:**
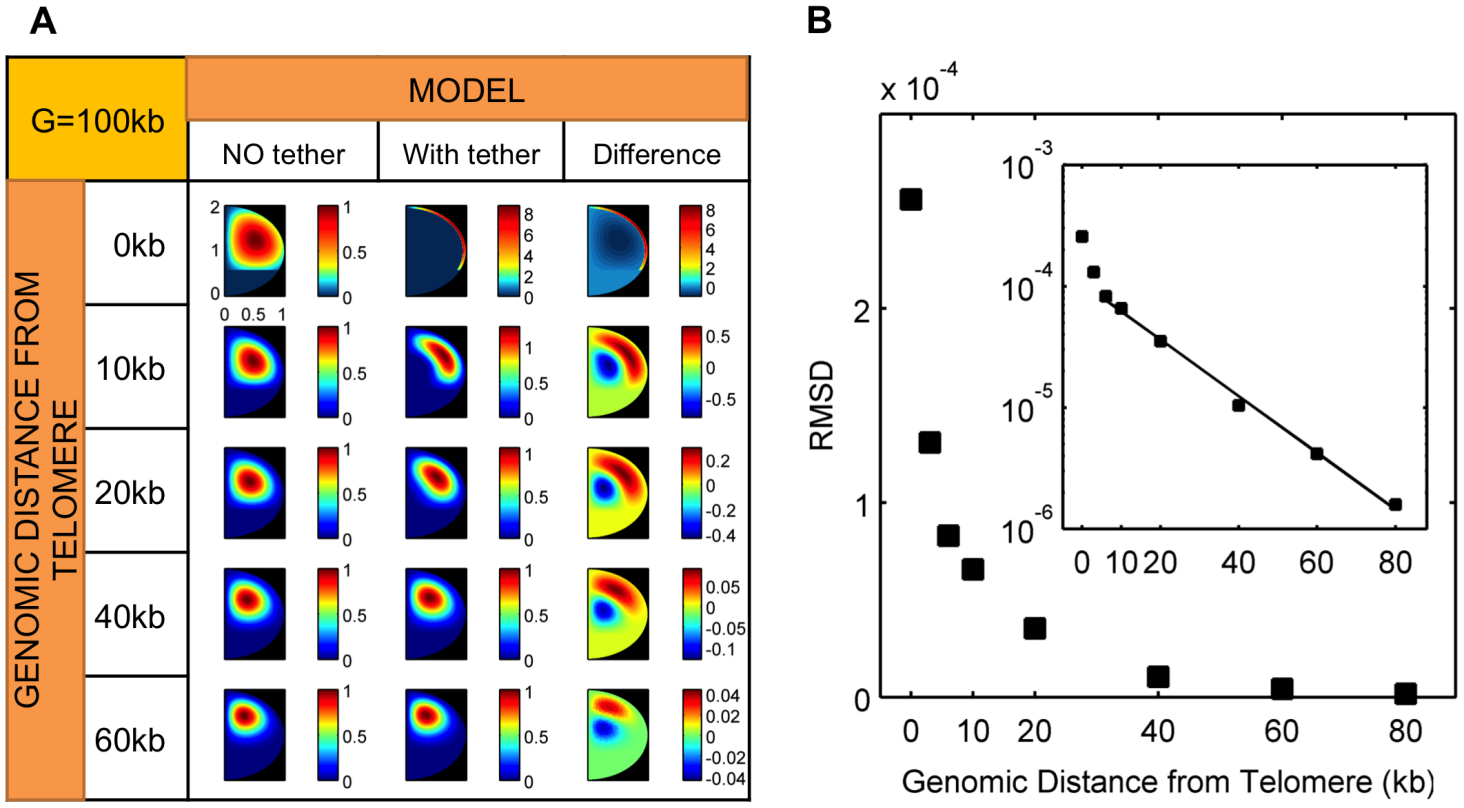
The effect of telomere tethering on gene positioning. A) Heat maps of the probability distributions for the position of genetic loci within the nucleus. The genes are located along a 100 kb chromosome arm at distances 0 kb, 10 kb, 20 kb, 40 kb and 60 kb away from the telomere. The probability distribution is projected to a plane that contains the north-south direction defined by the SPB and the nucleolus position, respectively (Figure 1). The relative probability density (normalized by the maximum) is shown for one half the nuclear sphere while the other half is equivalent by symmetry. For each gene, we show its spatial distribution when the telomere is attached to the nuclear envelope, and when the telomere is not attached. The “difference” heat maps were calculated by subtracting the “no tether” heat map from the “with tether” heat map – i.e. they show the change in the spatial distribution of the gene upon attachment of the telomere to the nuclear envelope. B) The root-mean-square of the probability difference (RMSDs) map quickly decays as the gene is moved away from the telomere. For all genetic loci, except the ones at 0 and 3 kb away from the telomere, the decay of the RMSD with increasing distance from the telomere is roughly exponential with a characteristic length of 20 kb. (The best fitting line shown in the figure is fit to all points except the point at 0 and 3 kb.) When calculating RMSDs, we do not apply the normalization mentioned above in which the maximum probability density of each “no tether” heat map is assigned a value of 1. Rather, we use the absolute probabilities for each pixel when subtracting the “no tether” heat maps from the “with tether” heat maps to obtain the “difference” heat maps.

Using the random-walk polymer model of yeast chromosomes, we compute the probability distribution of positions of a particular Kuhn segment in the polymer chain within the nucleus (see Methods), which represents the distribution of locations of a particular gene. To ascertain theoretically the effect of telomere tethering on the spatial organization of genes within the yeast nucleus, we computed this probability distribution in the presence and in the absence of a telomere tether. In Figure 2A, we juxtapose the “no tether” and “with tether” probability distributions for the spatial positioning of five genes located 0–60 kb away from the telomere on a 100 kb-long chromosome arm.

To quantify the effect of telomere tethering on gene positioning, we compute the root-mean-square of the difference (RMSD) between the two probability distributions (Figure 2B). We find that the effect of telomere tethering on gene positioning is most significant for genes adjacent to the telomere, and the effect decreases with increasing distance from the telomere. Specifically, the RMSD decreases faster than exponential for distances less than about 10 kb. For genes located more than 10 kb from the telomere, we find an exponential decrease in the magnitude of the effect with a decay length of about 20 kb. Repeating this analysis for chromosome arms that are 200 kb in length leads to the same conclusion (Figure S1). Our results are qualitatively consistent with previous experimental studies that concluded that disruption of tethering only affects subtelomeric regions of yeast chromosomes [34], [38], [66].

### Effect of telomere tethering on the positioning of the HML locus on chromosome III

Our polymer model calculations predict that telomere tethering has little effect on the positioning of genes that are not in the immediate vicinity of the telomere. To test this prediction we measured *in vivo* the position of the *HML* locus, which is located on the left arm of chromosomes III between 11 kb and 14 kb from the telomere [67]. We measure the positioning of this locus with respect to the SPB, in the presence and the absence of the telomere tether, and compare our measurements to predictions from theory. Furthermore, we construct a yeast strain where the *HML* locus itself is tethered (in addition to the telomere tether), with the expectation that this will have a large effect on its positioning. Our experiments confirm this qualitative expectation and also find good quantitative agreement between the theoretically predicted *HML*-SPB distance distribution and the measured one.

#### i. Theory

**Figure 3:**
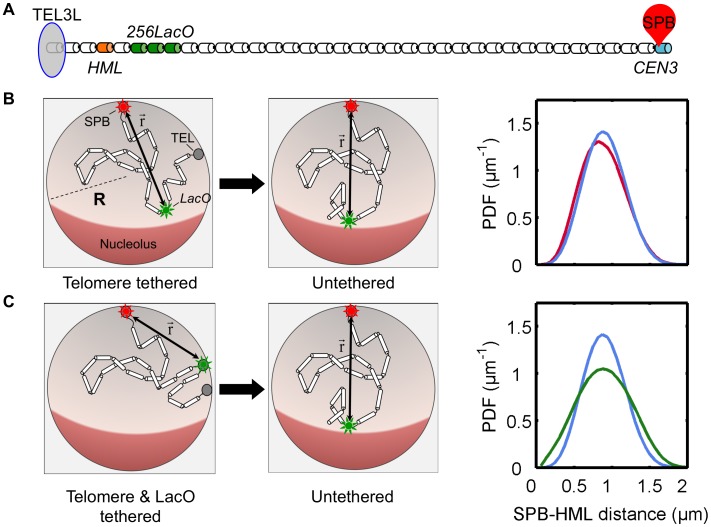
The effect of telomere tethering on the organization of chromosome III in yeast. A) Polymer model of the left arm of chromosome III in yeast is shown as a series of Kuhn segments, each containing 3.3 kb of DNA. 37 segments are joined together to represent 122 kb long chain which account for the yeast chromosome III with an additional ∼10 kb long LacO array (green) inserted proximal to the *HML* (orange). SPB (red balloon) is attached to the centromere (blue) locus. The left telomere (gray oval) of chromosome III is represented with a single Kuhn segment. B) Schematic diagrams of polymer configurations used in our theoretical calculations are shown in the first and second columns. The only difference between the two is the presence of absence of the telomere tether. In the third column we show the theoretically computed probability distributions for the distances between the SPB and *HML* proximal site, in the presence (red line) and absence (blue line) of the telomere tether. C) Schematic diagrams of the polymer models – same as B, are shown in the first (telomere and *HML* tethered) and second (untethered) columns. Theoretical probability density functions of distances between the SPB and *HML* proximal site computed from the polymer model of the left arm of chromosome III, with (green) and without (blue) tethers, one at the *HML* location and the other at telomere are shown in the third column.

In order to provide a theoretical prediction that we can test experimentally, we compute the distribution of distances between the SPB and an *HML* proximal site (corresponding to the location of the fluorescent marker in our experiments, the center of which is ∼6.5 kb from *HML*). In our computations the centromere is taken to be 50 nm away from the SPB, corresponding to the approximate length of the microtubule tether between the SPB and centromere [64], [65]. We model the left arm of chromosome III as a random walk polymer chain 122 kb in length, with a 20 kb long polymer segment between the telomere and the fluorescent marker for *HML* (Figure 3A). Both of these lengths take into account the size of the inserted operator array (10 kb) that was used in experiments to fluorescently tag *HML*. From our polymer-model calculations, we predict a small change in the distribution of distances between *HML* and SPB when the telomere is released from the nuclear membrane (Figure 3B). Somewhat counter intuitively the distribution of *HML-SPB* distances is predicted to slightly narrow upon release of the tether.

In Figure 3C we show the theoretical prediction for the distribution of distances between the SPB and a nuclear membrane-bound *HML*-proximal site (green curve). We use the same polymer parameters for the left arm of chromosome III as for the wild-type situation shown in Figure 3B, but we include an additional tethering interaction at the *HML*-proximal site. In our computations we assume that the probability that the HML-proximal site is tethered is 0.68, which is an estimate based on published data on the localization of the *LacO*-bound LacI-FFAT-GFP fusion protein within the yeast nucleus [40] (see Text S1). According to the polymer model, the SPB-*HML* distance distribution in this case is significantly affected by the removal of the two tethering interactions (blue curve), unlike what we concluded for the wild-type case when only the telomere is tethered to the nuclear periphery (Figure 3B).

#### ii. Experiments

**Figure 4:**
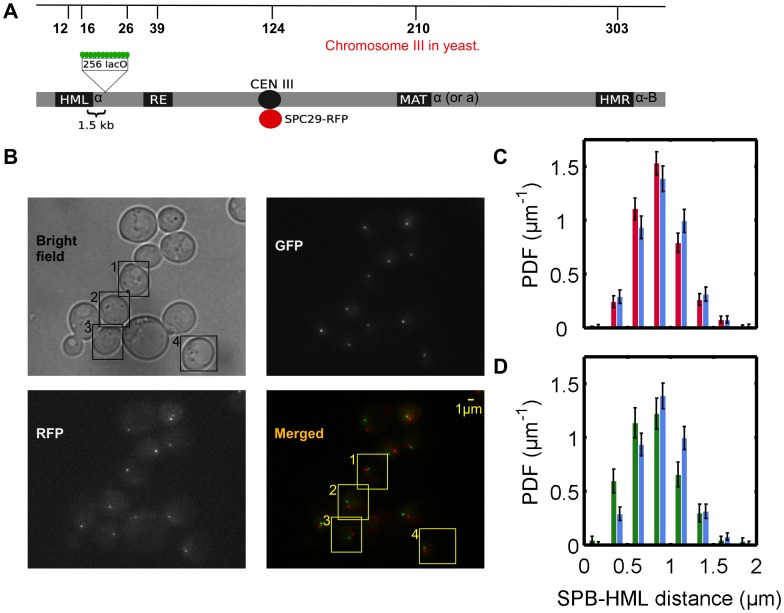
Quantitative fluorescent microscopy of the spindle pole body (SPB) and an *HML* proximal locus. A) Schematic view of budding yeast chromosome III (top line indicates the distance of each locus from the left telomere end in kb). 256 tandem repeats of Lac operators are inserted at a site 1.5 kb proximal to HML. Expression of GFP-fused to LacI or LacI-FFAT marks the locus in the proximity of HML. SPB component SPC29 is fused with RFP. B) Representative wide field microscopy images of yeast strain YDB271 are shown; top left: bright field, top right: green channel, bottom left: red channel and bottom right: merged and pseudo colored view of fluorescence channels red and green (scale bar 1 micrometer). Unbudded and G1 (cells with no duplicated SPB) – marked with boxes 1 to 4 – were selected to be analyzed for distance measurements. C) Experimental distributions of SPB-*HML* distances of 1,266 wild type (red bars) and 1,049 *yku80/esc1* double mutant (blue bars) cells. Error bars represent counting errors, which we estimated as twice the standard deviation of the number of measurements of distance that falls into each bin, calculated from the binomial distribution. The Kolmogorov-Smirnov test was used to check if these two data sets are indeed from a different distribution and it returned a p-value of 0.011. D) Experimental distributions of SPB-*HML* distances in case of 657 cells with *HML* tethering via LacI-FFAT-GFP bound to the *HML* proximal *LacO* array in addition to the wild type tethering of telomeres (green), and for 1049 *yku80*Δ *esc1*Δ double mutant cells (blue; same as in Figure 4C). Error bars are calculated as explained in C. The Kolmogorov-Smirnov test for these two data sets returns a p-value of 3.5×10^−9^, much lower than obtained by comparing the tethered and untethered distributions in Figure 4C.

To quantitatively test our theoretical predictions we made use of the wild-type yeast strain with an SPC29-RFP fusion protein that labels the SPB in red [68]. We also inserted a 256-tandem array of *LacO* sequences, which bind LacI-GFP, 1.5 kb proximal to the *HML* gene to label it green (Figure 4A) [69], [70]. We imaged cells in the G1 phase of the cell cycle to avoid the complications caused by the duplication of the SPB as well as by chromosome replication and subsequent chromosome condensation (Figure 4B). We measured the three-dimensional distance between the GFP and RFP markers to obtain the distribution of distances shown in Figure 4C and Figure 4D.

In order to determine the positioning of *HML* in the absence of telomere tethering during G1, fluorescence measurements were repeated using mutant strains with the *YKU80* and *ESC1* genes deleted thereby untethering the telomeric regions [21], [26], [71], [72]. Figure 4C shows the experimental distributions for the distances between the SPB and the *HML* proximal *LacO* array for these mutant strains. We observe a small shift in the probability distribution of distances between the SPB and *HML* when compared to the wild type distribution, in qualitative agreement with theory. (A detailed quantitative comparison of theoretical and experimental distributions is given below.)

Finally, we constructed a second mutant yeast strain with LacI-GFP fused to a nuclear membrane-targeting FFAT peptide motif containing two phenylalanines in an acidic tract, which binds to the integral ER membrane protein Scs2, and another yet-unidentified target on the nuclear membrane [40], [73]. Consequently, in these strains the *HML*-proximal locus is tethered to the nuclear membrane by the LacI-FFAT-GFP proteins bound at the *LacO* array. The measured distance distribution for this mutant is shown in Figure 4D. There we also compare it to the distance distribution measured in mutant strains in which both this synthetic tether and the telomere tether are absent and we see a much bigger shift of the distance distribution than in Figure 3C, as predicted by theory. Next we make quantitative comparisons between the measured and theoretically predicted distance distributions.

### Comparison of theory and experiments

**Figure 5:**
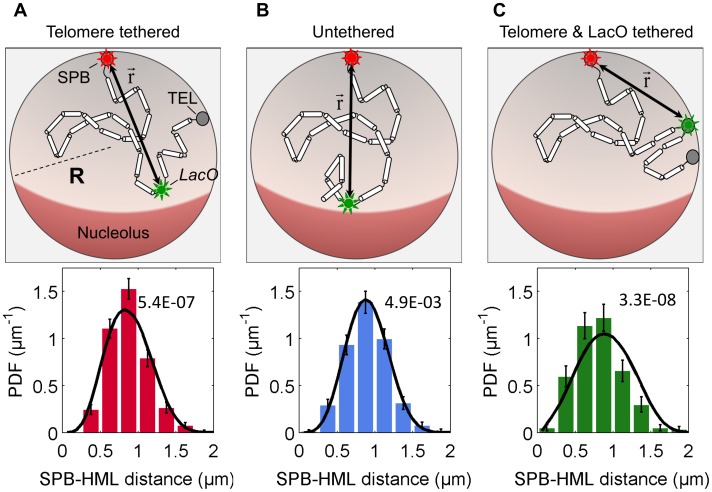
Comparison of theoretical and experimental distributions. Column (A): Telomere tethered – wild type; column (B): untethered - *yku80/esc1* double mutant; column (C): Telomere and *LacO* tethered – mutant carrying LacI-FFAT-GFP. Top row: a schematic diagram of the polymer models used for each strain. Bottom row: comparison of the experimental PDFs for wild type (red), *yku80/esc1* double mutant (blue), and mutant carrying LacI-FFAT-GFP (green) cells, and the corresponding theoretical PDFs (black curves in each graph). The parameters of the model are given in Table 1. The p-values for the one-sample Kolmogorov-Smirnov test that compares the experimental and corresponding theoretical distributions, are 5.4×10^−7^ for the wild type, 4.9×10^−3^ for untethered telomere mutant, and 3.3×10^−8^ for the *HML*-bound mutant (these p-values are also shown in the plots).

In Figure 5 (and Figure S2), we show a comparison of our theoretical distance distributions and those we experimentally obtained for the wild-type yeast cells and the two mutants described in the in the previous section. Notably, all three theoretical distributions were computed with the same model parameters (see Table 1) obtained from a maximum likelihood fit of all the data simultaneously (see Text S1). When extracting parameter values using the maximum likelihood method they were constrained to lie within the ranges reported previously [12], [13], [74].

The comparison between the theoretically and experimentally obtained distance distributions gives a small but still statistically significant discrepancy for the two strain where the chromosome is tethered at the telomere, or both at the telomere and at the HML-proximal locus (Figure 5A and Figure 5C respectively). The untethered mutant on the other hand shows excellent agreement between theory and experiment (Figure 5B). There can be a number of reasons for the observed discrepancy. One possibility is that the telomere of chromosome III is confined to a specific region of the nuclear envelope due to an interaction with some membrane-bound protein. Another one, which we think more likely, is that the probability of the telomere bound at the nuclear periphery is less than one, i.e., the tethering is not perfect. Both extensions of the model lead to a distance distribution that is sharper than what we have obtained with our simple polymer model, and would give better agreement with our experimental observations (at the price of introducing new parameters for which we have no independent experiments).

While the comparison between theory and experiment is not perfect we believe our combined experiments and theory give strong support for the conclusion that the positioning of only those genes that are within 10 kb of the telomere tether are affected by this tethering interaction between the chromosome and the nuclear periphery. It also provides further support for the random-walk polymer model of yeast chromosomes.

## Discussion

Three-dimensional chromosome organization in the yeast nucleus provides a powerful model system for understanding the spatial organization-function relationship for eukaryotic genomes. For yeast chromosomes, their spatial organization is described in quantitative detail by a random-walk polymer model that takes into account the tethering of the telomeres to the nuclear membrane and the centromeres to the spindle pole body (Figure 1). The key result of this paper is that telomere tethering to the nuclear periphery significantly affects the positioning of only subtelomeric genes, within ten kilobases from the telomere. We tested this prediction experimentally by measuring the positioning of the HML locus on chromosomes III under different tethering scenarios and found good agreement between theory and experiment. Our detailed comparisons between theory and experiments also serves as a quantitative test of the random-walk polymer model of yeast interphase chromosomes [53], [55].

### Effect of chromosome tethering on transcription and double strand break repair

Previous studies suggest a link between chromosome function and the tethering of chromosomes to the nuclear envelope. In budding yeast, positioning of genes in close proximity to telomeres causes transcriptional silencing [75], [76], on the other hand a reporter gene flanked by two functional *HML* silencers became desilenced when placed more than 200 kb from the telomeres [77]. It was also shown that transcriptional repression of the *HMR* gene occurs when it is artificially tethered to the nuclear envelope, despite *HMR* having a defective silencer sequence [37]. In contrast, other studies have shown that dynamic recruitment of genes to the nuclear pore complexes increases their transcriptional activity [8], [39], [40].

Experiments that address the nuclear positioning of subtelomeric loci revealed important functional roles related to genomic integrity. Louis et al. found a recombination barrier between sequences at telomeric and internal locations, which involves the yeast protein Ku80 [78]–[80]. In another study, the efficiency of double-strand break repair of two I–SceI cleavage sites inserted on the left arm of yeast chromosome XI 3.5 kb from the telomere was reduced in the absence of proper attachment at the nuclear envelope by disrupting the nuclear pore complex [38]. Moreover, recent work addressing the effect of nuclear organization on genome integrity revealed that tethering of telomeres and centromeres reduces the efficiency of DNA recombination between distant genomic loci [9].

If indeed the positioning of genes within the nucleus modulates their function, then our results suggest that only genes very close to the telomere (or centromere) will have this function strongly affected by telomere attachment. Interestingly, telomere proximal suppression of transcriptional activity of yeast loci has been observed for genes within 20 kb of the telomeres [76]. Should the cause of such transcriptional suppression be related to the genes' spatial positioning within the nucleus, our results may explain why the suppression occurs only for genes within 20 kb of the telomere: only the positioning of those genes is significantly influenced by the membrane-attachment of the telomere, so perhaps only these genes localize to the nuclear periphery enough to undergo transcriptional suppression.

The observations in the aforementioned studies suggest that there might be a link between chromosome tethering and function. This connection could be established more conclusively by determining whether the transcriptional activity or the propensity for recombination of subtelomeric loci is substantially affected by the removal of telomere tethering, or by introducing artificial membrane tethers close to genes of interest.

## Materials and Methods

### Yeast strains and plasmids

The yeast strains used in this study can be found in Table 2. All strains used were variants of YDB076 [70]. YDB076 was transformed with the PCR fragment of *SPC29-RFP-(KAN-MX)*, amplified from KBY5055 (a gift from Kerry Bloom), to construct YDB257, and next YDB257 was transformed with the NotI restriction fragment of pAG60 [81] to replace the KAN-MX cassette with a Ca-URA3-MX and construct YDB270. YDB271 was constructed by transforming YDB270 with NheI digest of pDB030 [70]. YDB276 was constructed by expressing *HO* by inducing YDB271 cells in galactose containing media to switch from *MATa* to *MATα*. YGM24 and YGM25 were created by replacing URA3-MX marker with NAT-MX cassette obtained from pJH1513 via NotI restriction digest and deleting *YKU80* using a BamHI/SalI restriction fragment from pJH1729, and by deleting *ESC1* using transformation of a PCR-amplified fragment obtained from genomic DNA of the Research Genetics strain collection on YDB276 and YDB271 background respectively. The strain carrying the FFAT binding domain inserted between LacI-GFP, YBA006, was constructed by transforming YDB270 with pBA001 cut with NheI. pBA001 was derived by subcloning a KAN-MX cassette, NotI restriction digest fragment from pJH1512, into the plasmid pGFP-FFAT-LacI (a gift from Jason Brickner) [40] cut with the same.

**Table 2:** Yeast strains used in this study.

Strain	Genotype
YDB076	*ho HMLα HMLprox::LacO(256)-LEU2 MATa HMRα-B ade1 ade3::GAL-HO leu2 trp1:hisG ura3-52*
YDB257	YDB076 with *Spc29-RFP-(KAN-MX)*
YDB270	YDB257 with *Spc29-RFP-(Ca-URA3-MX)*
YDB271	YDB270 with *HIS3::URA3pro-LacI-GFP-(KAN)*
YDB276	Same as YDB271 except *MATα*
YGM024	YDB276 except *Spc29-RFP-(Ca-NAT-MX) yku80::URA3 esc1::KAN*
YGM025	Same as YGM024 except *MATa*
YBA006	*YDB270 with HIS3::HIS3pro-LacI-FFAT-GFP-(KAN-MX)*
YBA007	Same as YBA006 except *MATα*

### Preparation of fixed cells

To maximize the number of cells that are in G1 phase of the cell cycle, cells were grown overnight to reach stationary phase. Stationary phase cells were counted and inoculated into fresh medium with final concentration 5×10^6^ cells/ml. Cultures were collected after 4 hrs and cells were fixed by addition of paraformaldehyde at a 2% final concentration for 10 minutes at room temperature. Following this, cells were pelleted and washed in 0.1 M potassium phosphate, pH 6.6 for 10 minutes at room temperature. Cells were pelleted a second time and resuspended in 35–50 µl of 0.1 M potassium phosphate, pH 6.6 and stored at 4°C before imaging at room temperature [70].

### Acquisition and processing of fixed cell images

Images of fixed cells were acquired on an Olympus BX41 wide field microscope equipped with a mercury lamp for epi-fluorescence, a Photometrics DV2 dual view apparatus for signal separation of red and green channels, and a Hamamatsu ORCA-R2 CCD camera for signal detection. 16 to 20 Z-sections were acquired at 0.2 µm steps using a 100X 1.4 NA Olympus U-PlanApo objective with 1×1 binning. Cells with buds, with multiple fluorescent spots of the same color and with deformed cell membrane were excluded from imaging to protect sample uniformity.

Cells were imaged using a GFP-DsRed dichromatic excitation/emission filter cube set with exposure time of 0.3 s. Images were recorded with Metamorph software (Molecular Devices) and analyzed with the ImageJ plugin, SpotDistance (EPFL Biomedical Imaging Group) [82], with pixel sizes 64.5 nm, 64.5 nm and 200 nm for x, y and z axes respectively to calculate the three-dimensional distances between the fluorescent spots. Corresponding distance measurements are given in Data S1.

### Random walk simulation and selection of model parameters

We used custom MATLAB scripts to simulate the yeast chromosomes as confined and tethered random walk chains; see Figure 1. This model required six parameters that are given in Table 1. For a given set of parameter values, at least one million random walk polymer chains representing the left arm of chromosome III were generated. Each random walk polymer configuration was confined to a sphere of radius R, representing the nucleus. Each run started at a random position within the nucleus, which was chosen at a fixed distance from the north pole given by the length of the microtubule between the SPB and centromere. Then steps of the random walk all equal to the Kuhn length were taken in randomly chosen directions. *N = G/GK* gives the total number of steps, where *G* is the genomic length of the chromosome arm and *G_K_* the Kuhn length in base pairs. Only random walks that satisfied the constraints that they did not leave the confines of the nucleus and that they ended at the nuclear periphery (for telomere tethered chromosomes) were kept. For each valid configuration generated in this way the position of the Kuhn segment representing the *HML* locus was recorded. To determine the optimal parameter values for our model (Table 1), we performed maximum likelihood estimation based on all the data we collected. The ranges of parameter values examined in the maximum likelihood procedure were based on previously reported experimental (details of the maximum likelihood estimation are given in the Text S1). To test our random walk simulations we compared the results for a simplified model, which does not have the nucleolus, with analytic results based on the Green's function for the diffusion equation in a sphere, and found excellent agreement.

### Computing heat maps for the spatial positioning of genes

Following the parameter estimation (Table 1), we simulated the chromosome arms of different length with or without a nuclear membrane attachment. We recorded the three-dimensional coordinates of seven genetic loci that are located 0 kb, 3 kb, 6 kb, 10 kb, 20 kb, 40 kb and 60 kb respectively from the telomere. Because of the radial symmetry of the model, we reduced the 3D coordinates to only two coordinates: (1) the z-coordinate, where the z-axis runs from the spindle pole body to the opposite end of the nucleus, and (2) the radial distance from the locus to the z-axis – i.e. the magnitude of the position-vector projection onto the x-y plane. We subdivided this 2D coordinate plane into 10 nm by 10 nm bins and calculated the probability of finding the gene in each of the bins.

## Supporting Information

Figure S1 **Effect of tethering on positioning of loci on a 200 kb length arm.** A) Heat maps showing the probability distribution of the position of different loci, computed with (column 2) and without (column 1) a telomere tether at the end of the chromosome arm. Colors from red to blue represent probability values from high to low, respectively. The differences between column 2 and column 1 are displayed in column 3. B) The RMS of the difference between the heat maps that are simulated in the presence and absence of a telomere tether shown on a linear and on a semi logarithmic plot (inset). The line in the inset is obtained from a linear least-squares fit, indicating an exponential fall-off.
(TIF)

Figure S2 **Comparison of theoretical and experimental cumulative distributions.** Column (A): Telomere tethered – wild type; column (B): untethered - *yku80/esc1* double mutant; column (C): Telomere and *LacO* tethered – mutant carrying LacI-FFAT-GFP. Top row: a schematic diagram of the polymer models used for each strain. Bottom row: comparison of the experimental cumulative distribution function (CDFs) (dashed lines) and the theoretical CDFs (solid lines).
(TIF)

Text S1 **Supplementary information.** Detailed explanation of (i) parameter selection using maximum likelihood estimation and (ii) computing the membrane association of the FFAT fusion protein.
(DOCX)

File S1 **Supplementary data.** Experimental three-dimensional distances between *HML* proximal insert and SPB.
(XLSX)
