# Supplementary figures and images for: Effect of Chromosome Tethering on Nuclear Organization in Yeast
Source: PLoS One. 2014 Jul 14;9(7):e102474. doi: 10.1371/journal.pone.0102474 (PMC4096926; doi:10.1371/journal.pone.0102474)

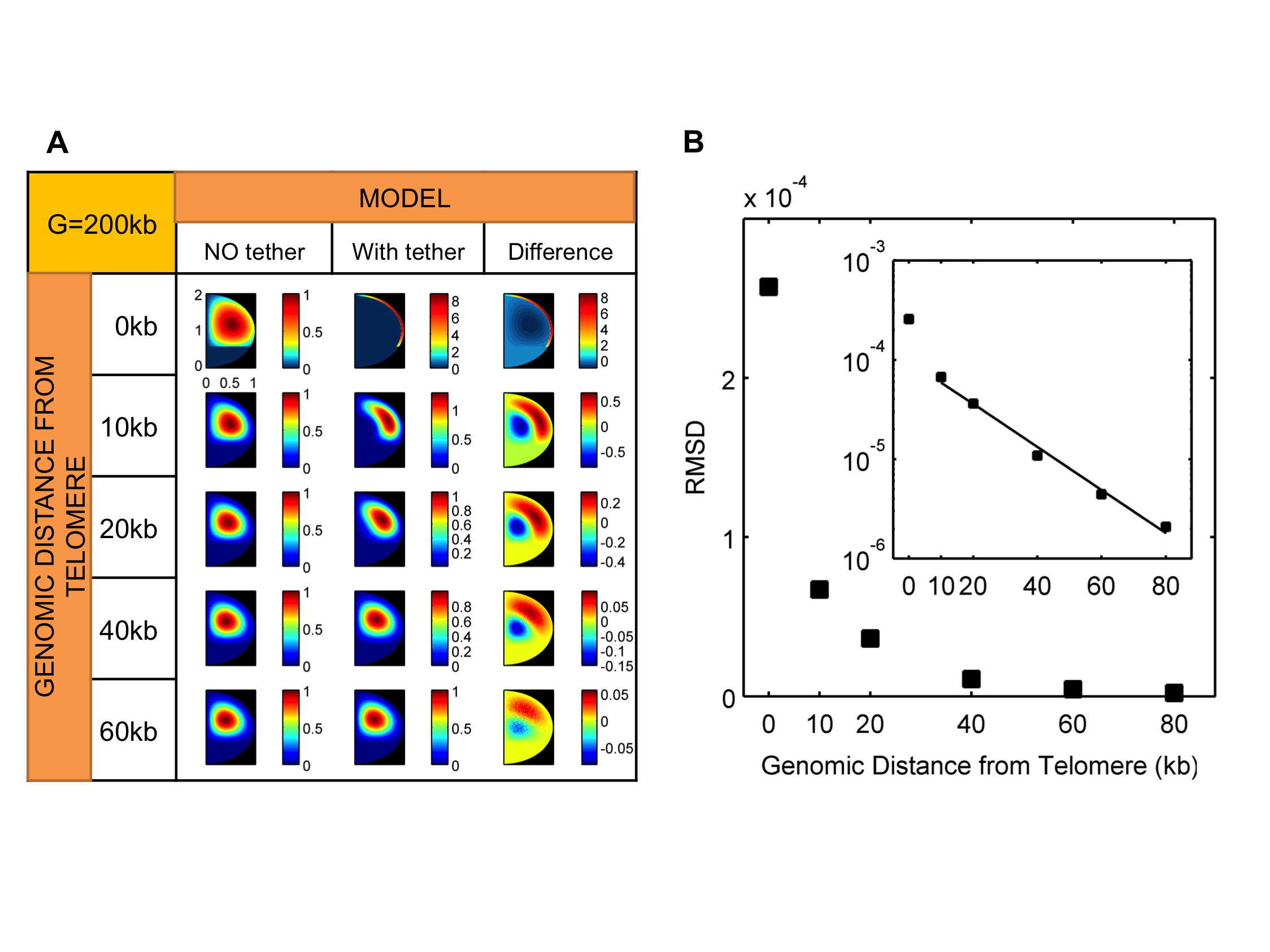

Supplement: Figure S1 — Effect of tethering on positioning of loci on a 200 kb length arm. A) Heat maps showing the probability distribution of the position of different loci, computed with (column 2) and without (column 1) a telomere tether at the end of the chromosome arm. Colors from red to blue represent probability values from high to low, respectively. The differences between column 2 and column 1 are displayed in column 3. B) The RMS of the difference between the heat maps that are simulated in the presence and absence of a telomere tether shown on a linear and on a semi logarithmic plot (inset). The line in the inset is obtained from a linear least-squares fit, indicating an exponential fall-off. (TIF) [file pone.0102474.s001.tif]

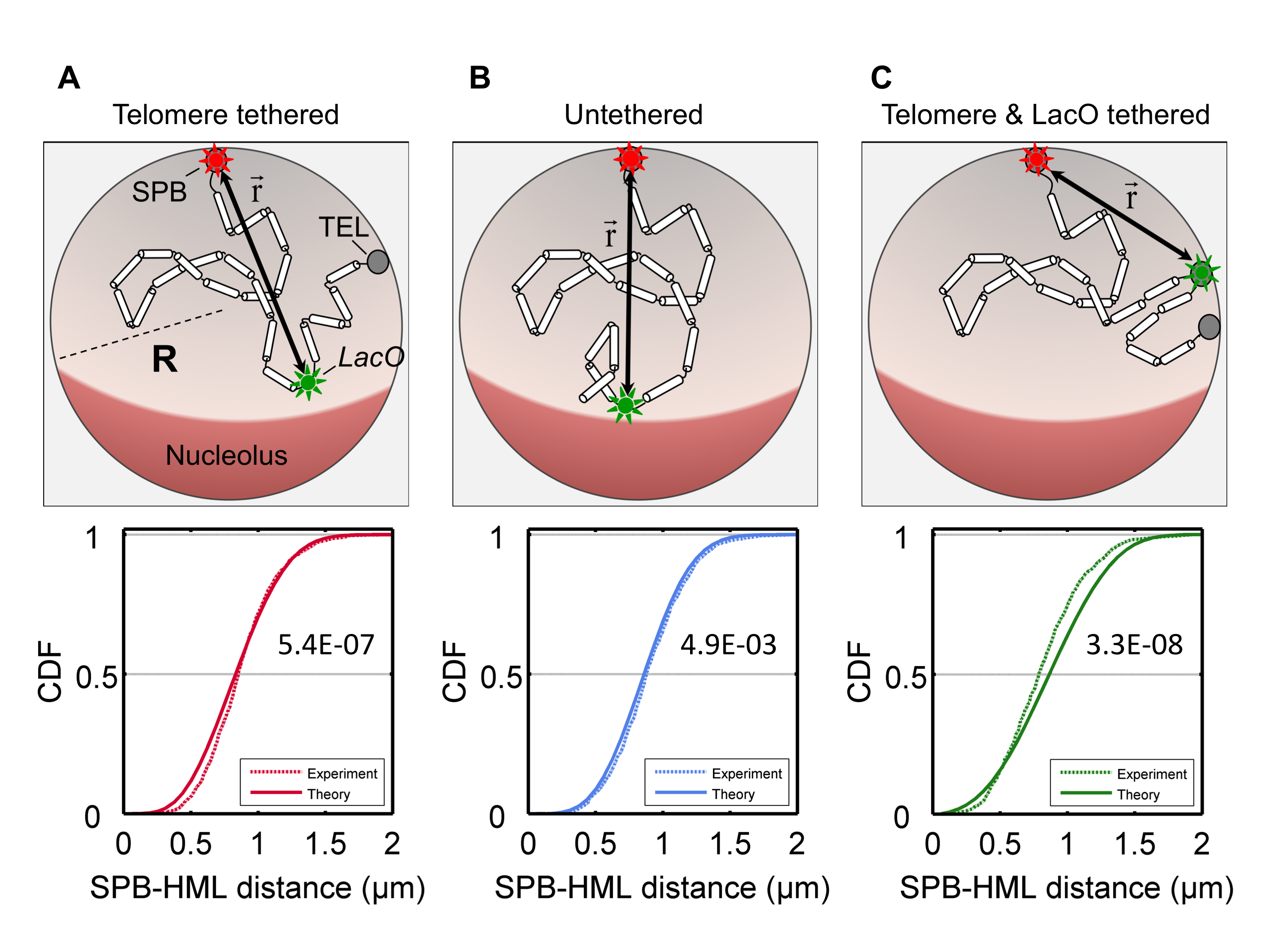

Supplement: Figure S2 — Comparison of theoretical and experimental cumulative distributions. Column (A): Telomere tethered – wild type; column (B): untethered - yku80/esc1 double mutant; column (C): Telomere and LacO tethered – mutant carrying LacI-FFAT-GFP. Top row: a schematic diagram of the polymer models used for each strain. Bottom row: comparison of the experimental cumulative distribution function (CDFs) (dashed lines) and the theoretical CDFs (solid lines). (TIF) [file pone.0102474.s002.tif]
