# Supplementary material for: Effect of Chromosome Tethering on Nuclear Organization in Yeast
Source: PLoS One. 2014 Jul 14;9(7):e102474. doi: 10.1371/journal.pone.0102474 (PMC4096926; doi:10.1371/journal.pone.0102474)
Supplement: Text S1 — Supplementary information. Detailed explanation of (i) parameter selection using maximum likelihood estimation and (ii) computing the membrane association of the FFAT fusion protein. (DOCX) [file pone.0102474.s003.docx]

# SUPPLEMENTARY INFORMATION

## Parameter selection using maximum likelihood estimation

To estimate the optimal parameter set for the model given our experimental data, we performed maximum likelihood estimation. We selected a set of values for our model parameters, simulated the chromosome arm for the three yeast strains, computed the three corresponding probability density functions for SPB-HML distances, and used these PDF’s to determine the log-likelihood of the parameter set. The above procedure was repeated for different parameter values spanning the range of values reported by previous experimental studies (see Table 1 in the main text). After computing the log-likelihoods for parameter sets from across the entire range of previously reported values, parameter sets were selected from a smaller region of parameter space where the highest log-likelihoods were found. Within this subset of parameter space, the values for the mean nuclear radius, standard deviation of the nuclear radius, nuclear volume percentage, and chromosome extension parameter were varied in increments of 0.05 μm, 0.01 μm, 5%, and 1 μm^2^/Mb, respectively. For all calculations in this paper, we use the parameter set (see main text 1) that maximizes the log-likelihood function *L* (below).

For a particular parameter set, the model’s SPB-*HML* distance distributions predict a PDF value for each experimentally measured distance. The log-likelihood function *L* is given by

$$L=\sum_{i=1}^{N_{WT}} ln{PDF}_{WT}(i)+\sum_{i=1}^{N_{UNTETH}} ln{PDF}_{UNTETH}(i)+\sum_{i=1}^{N_{FFAT}} ln{PDF}_{FFAT}(i)$$

where *PDF_WT_*(*i*) is the model’s predicted PDF value for the *i*^th^ wild-type experimentally-measured SPB-*HML* distance. The microscope’s resolution is 220 nm, so we assign a predicted PDF value to each experimentally measured distance by calculating the model’s average PDF value between -110 nm and +110 nm of the experimental distance reported by the image analysis software. *N_WT_* is the total number of experimentally measured SPB-*HML* distances using the wild-type strain. The subscript “UNTETH” is for the strain with the untethered telomere, and the subscript “FFAT” is for the strain containing the LacI-FFAT-GFP fusion protein.

The percentage of the nuclear volume occupied by the nucleolus, reported in Table 1 of the main text, indicates that the nucleolus occupies a fixed volume equal to 20% of a nucleus of radius 0.95 μm (i.e. nucleolar volume = 0.72 μm^3^). While the model includes a variable radius, the absolute nucleolar volume is kept constant – i.e. for each run of the simulation, whatever nuclear radius is randomly selected from a normal distribution with a mean of 0.95 μm and a standard deviation of 0.09 μm, the height of the spherical cap representing the nucleolus is adjusted to obtain a volume of 0.72 μm^3^. The constant volume of the nucleolus is apparent from examination of data from Berger et al. [1] where a fixed nucleolar volume is a more likely model than a nucleolar volume that scales with the nuclear volume.

The model contains three additional parameters that have little effect on the SPB-*HML* distance distribution. These parameters are (1) the distance from the centromere to the spindle pole body, (2) the Kuhn length, and (3) the thickness of the region at the nuclear periphery in which the simulated telomere must be located for the telomere to be considered bound to the nuclear membrane.

## Computing the membrane association of the FFAT fusion protein

In the yeast strain YBA006, the fusion protein LacI-FFAT-GFP has probability P of binding the *LacO* array to the nuclear membrane – i.e. in a population of YBA006 cells, P of the cells have the *LacO* array bound to the nuclear envelope by the fusion protein. To model YBA006, it is necessary to determine P, which we calculate using data from Figure 5B of Brickner and Walter 2004 [2].

Brickner et al. do not measure P but rather measure the fraction F of cells in which the distance between the *LacO* array and the nuclear envelope is no more than 10% of the nuclear radius. F values for cells with LacI-FFAT-GFP under different growth conditions are reported in bars 1 and 2 of Figure 5B. If the *LacO* array is found in this region (now referred to as the “10% region”), then LacI-FFAT-GFP is binding the *LacO* array to the nuclear membrane, or LacI-FFAT-GFP is not bound to the nuclear membrane but the *LacO* array is occupying this region nonetheless (the *LacO* array may have wandered into this region by chance, or the *INO1* gene may be bound to the nuclear membrane, as will be discussed below). We can express this as an equation:

$$F = P + (1-P)*C$$

where C is the probability of the *LacO* array occupying the 10% region when LacI-FFAT-GFP is not bound to the nuclear membrane. We will make the following assumption: C is equal to the probability in wild-type cells that the *LacO* array occupies the 10% region. This probability for wild-type cells is reported for different growth conditions in bars 5 and 6 of Figure 5. Note that different growth conditions could change the *LacO* array’s localization probabilities, so the cells with LacI-FFAT-GFP and the wild-type cells must have identical growth conditions for our assumption to hold. For example, inositol triggers the dissociation of the *INO1* gene (located adjacent to the *LacO* array in the strain used in [2]) from the integral membrane protein Scs2. Clearly, the presence or absence of inositol affects the frequency of localization to the nuclear membrane. For our calculation of P, it does not matter whether we use data from the cells with inositol or those without, so long as the values of F and C that we use come from only one growth condition or the other.

For the case in which inositol is present, F = 0.85 ± 0.04 (bar 1), and C = 0.32 ± 0.04 (bar 5). Using these values in equation 1 and solving for P, we obtain P = 0.78 ± 0.10. For the condition in which inositol is absent, F = 0.76 ± 0.05 (bar 2), and C = 0.52 ± 0.05 (bar 6), which yields P = 0.50 ± 0.17. (Note that the error that we report is the standard deviation of the binomial distribution, which is slightly greater than the estimates of the standard error of the mean reported in [2].) Weighting the two values for P above by their corresponding errors, we find that P = 0.68 ± 0.09.

## Supplemental References

1. Berger AB, Cabal GG, Fabre E, Duong T, Buc H, et al. (2008) High-resolution statistical mapping reveals gene territories in live yeast. Nat Methods 5: 1031–1037.

2. Brickner JH, Walter P (2004) Gene recruitment of the activated INO1 locus to the nuclear membrane. PLoS Biol 2: e342.
